# 

**Published:** 1883-01

**Authors:** 


Vol. XXXVII. JANUARY, 1883. No. 1.

Feb 5

### THE
### Dental Register.

### A MONTHLY JOURNAL,

DEVOTED TO THE INTERESTS OF THE PROFESSION.

EDITED BY J. TAFT, D.D.S.

PUBLISHED BY

SPENCER & CROCKER,

OHIO DENTAL DEPOT,

Nos. 117, 119, 121 WEST FIFTH STREET, CINCINNATI, OHIO.

TERMS:—$2,00 Per Annum, in Advance. Single Copies, 25 cts.

